# Preparation of SLN-containing Thermoresponsive In-situ Forming Gel as a Controlled Nanoparticle Delivery System and Investigating its Rheological, Thermal and Erosion Behavior

**Published:** 2015

**Authors:** Golnar Dorraj, Hamid Reza Moghimi

**Affiliations:** Department of Pharmaceutics and Nanotechnology, School of Pharmacy, Shahid Beheshti University of Medical Sciences, Tehran, Iran.

**Keywords:** Solid lipid nanoparticle (SLN), In-situ forming gel, Controlled release, Rheology, Erosion.

## Abstract

Various nanoparticles have been investigated as novel drug delivery systems, including solid lipid nanoparticles (SLNs). Due to their rapid clearance from systemic circulation, nanoparticles do not provide sustained action in most cases. Different strategies have been employed to overcome this problem. In this direction, the present study introduces erodible in-situ forming gel systems as potential vehicles for prolonged release of SLNs.

SLNs were prepared by solidification of an oil-in-water microemulsion containing stearic acid, surfactants and co-surfactants. Nanoparticles were then dispersed in a thermosensitive Poloxamer 407 aqueous solution (sol) at 4 °C and their effects on gel forming ability, sol-gel transition and rheological behavior of the system were investigated over 5-50 °C. Thermal behavior of the system was investigated by differential scanning calorimetry too. Erosion rate of the gel in the presence and absence of SLN was measured by gravimetric method. Integrity of SLNs in the system was investigated by scanning electron microscopy (SEM) and particle size analysis.

SLN showed particle size and zeta potential of 130 ± 1.39 nm and - 44 ± 2.1 mV respectively. Particle size analysis and SEM studies after gel erosion revealed presence of intact SLN in the hydrogel. SLN reduced erosion rate of Poloxamer gel and increased its sol-gel transition temperature from 26 to 29 °C. However, gelling kinetic did not change significantly after addition of SLN. Damping factor <1 indicated stability of the SLN-containing system.

Present results indicate potential of sol-gel systems for controlled nanoparticle delivery and show that SLN affects properties of the system.

## Introduction

Novel controlled drug delivery systems are designed to deliver drugs at predetermined rates for predefined periods at target organs and overcome the limitations of conventional formulations, to diminish side effects and improve the life quality of patients (1). In recent years, various nanoparticles have been investigated as novel carriers for controlled drug delivery, including solid lipid nanoparticles (SLNs) (2), liposomes (3), nanoemulsions (4), polymeric nanospheres (5) and *etc*. These nanocarriers are taken up by cells more easily than larger systems and are shown to be promising in cellular drug delivery, gene-delivery, drug delivery to CNS and treating chronic diseases such as cancer (6).

One of the main disadvantages of nanoparticles is their rapid clearance from the blood circulation by reticuloendothelial system (RES). As a result, they will accumulate mainly in liver, spleen and lymph nodes. Their rapid clearance will cause a temporal (time-based) delivery problem; hence require frequent application of these particles. Uptake by RES system causes another challenge, lack of targeting, as well (7). Various strategies have been employed to overcome such problems including PEGylation and size and surface modifications of nanoparticles (8-10), however, in spite of all these techniques, the problem is yet to be solved completely.

As an alternative approach, our group has introduced application of injectable thermoresponsive in-situ forming sol-gel systems as a controlled release matrix for prolonged release of liposomes after intraperitoneal (IP) injection (11, 12). These in-situ forming systems are injected as aqueous solutions (sol) that set to form a gel at the target site. Blood concentration profile of these carriers indicated prolonged release of liposomes from hydrogel in a two-phase manner including a descending trend in early hours regarding gel formation followed by an ascending trend due to gel disappearance by time. Liposomal hydrogel also affected biodistribution of the marker, indicating release of intact liposomes from the gel after IP injection (12).

Present investigation aims to apply the same idea for SLNs. SLNs are widely investigated as parenteral pharmaceutical dosage forms because they offer many advantages such as low toxicity (due to their physiological lipid composition), biodegradable properties (13), possibility of incorporation of both hydrophilic and lipophilic drugs, enhanced drug stability and ease of lyophilization and sterilization (14). These particles can also improve drug delivery to brain (15). 

Among different injectable in-situ forming gels, this investigation employs biodegradable thermoreversible hydrogels that are more versatile than other systems. These systems respond to temperature changes as a physical transition and, unlike other forms, do not require use of organic solvents, chemical cross-linking reactions or externally applied devices (*e.g*. photo polymerization) (16, 17).

Lutrol^®^ F127 (also called Poloxamer 407) is one of the widely used thermoreversible hydrogels. This polymer is a triblock non-ionic, amphiphilic ABA-type polymer consisting of a single polyoxypropylene (PO) and two polyoxyethylene (EO) blocks. It contains 70 % PO, usually regarded as nontoxic (18, 19) and biocompatible and has been applied for localized or systematic depot drug delivery such as ophthalmic (20), intramuscular, IP, and subcutaneous injections (21). 

Most scientific investigations have approved the potential of Poloxamer 407 gels for sustained topical drug delivery for therapeutics such as anticancer (22), anti-inflammatory (23), antibiotic (24) and burn healing agents (25). These are also used as injectable dosage forms for controlled release of peptides and proteins (26).

In this study, Poloxamer 407 aqueous systems loaded with different amounts of SLN, optimized and evaluated as novel prolonged release system for injectable nanoparticles.

## Experimental

*Materials and methods*

Stearic acid was purchased from Kirish Pharma GmbH (Salzgitter, Germany); Lipoid E80 (egg phosphatidyl choline 80%) was obtained from Lipoid (Ludwigshafen, Germany), taurocholate sodium salt and Tween 80 were purchased from Fluka (Buchs, Switzerland). Poloxamer 407 (Lutrol^®^ F127) was obtained from BASF (Ludwigshafen, Germany). All other chemicals used were of analytical grade.

*Preparation of SLNs*

SLNs were prepared by diluting a warm oil-in-water (o/w) microemulsion in cold water using a method employed by Gasco (27) and formulation introduced by Bucca *et al.* (28). The microemulsion composed of stearic acid (0.70 mmol) melted at 70 °C as lipidic phase, egg phosphatidyl choline (0.14 mmol) as surfactant, taurocholate sodium salt as a cosurfactant (0.69 mmol), and distilled deionized filtered water (110.0 mmol) as a continuous phase (28). Hot (70 °C) aqueous surfactant solution was added to hot lipid phase dropwise, while stirring at 70 °C in water bath (Dorsa, Iran), until a clear homogenous microemulsion was obtained. SLNs were solidified by dispersing the warm microemulsion (at about 70 °C) in cold distilled water containing Tween 80 (0.5% w/w) as stabilizer at a ratio of 1:10 (microemulsion: cold water, v/v) under mechanical stirring (Heidolf, RZR1, Germany). The concentration of stabilizer was obtained from investigating the effects of surfactant on the size and foaming of the system (data are not provided here). Stirring carried out at 2500 rpm for 2 h. The samples were then purified by centrifugal filtration using Vivaspin 2 with MWCO of 5000 Da (Sartorius AG, Germany). Purified samples were stored in refrigerator until use.

*Preparation of SLN-loaded sol-gel system*

Poloxamer solutions were prepared according to the “cold method” described by Schmolka (25). Briefly, Poloxamer flakes were added to chill deionized water (5 °C) slowly over a period of about 2-3 minutes with gentle stirring by magnetic stirrer; RH-Basic 2 (IKA, Germany). The polymer solution was then placed in a refrigerator for a period of approximately 24 hours or until all the Poloxamer 407 had dissolved and a clear solution was obtained. For incorporation of SLN into Poloxamer solution and also to investigate the effects of SLN dispersion on the gelling properties of the system, required amounts of Poloxamer flakes was added to different mixtures of SLN dispersion/cold water with SLN dispersion contents of 0-80 mL and total Poloxamer content of 20% (w/v). Systems were placed in refrigerator until all polymers had dissolved. The capacity of this in-situ forming system for SLN loading was evaluated by observing gel consistency and gel clarity.

*Particle size and zeta potential analysis*

The size of nanoparticle dispersion was measured by laser diffraction using a Malvern Hydro 2000 SM particle size analyzer (Malvern Instrument, UK). Zeta potential of the lipid nanoparticles was measured by a Malvern Zetasizer Nano Instrument (NanoZS, Malvern, UK). Samples were diluted in distilled water before measurements. Results were reported as mean and standard deviation of three independent measurements performed at 25 °C.

*Electron microscopy*

Morphology and particle stability of SLNs were analyzed using scanning electron microscopy (SEM) (Philips, XL30). One drop of freshly prepared SLN dispersion with or without polymer was deposited on sample stub covered with carbon tab. The samples were then air-dried and covered with gold. To avoid SLN deformation or melting, measurements were performed at low voltages (10 kV).

*Phospholipid assay*

To evaluate SLN content of samples and lipid recovery (yield) of the preparation, the phospholipid content of SLN dispersions, as an indicator of the total lipid content, was measured by Stewart method (29). This colorimetric method is based on the formation of a colored complex (with λ max of 485 nm) of phospholipids and ammonium ferrothiocyanate. Calibration graph of lipid in chloroform was obtained over 0.3 to 0.8 mg and samples were analyzed against blanks (samples without lipid) at 485 nm using a UV-Visible spectrophotometer CE 2021 series 2000 (Cecil, United Kingdom).

*Differential scanning calorimetry (DSC)*

Thermal behavior of SLN dispersions was evaluated with a Shimadzu 60 DSC system (Shimadzu, Japan). Samples of 10–20 mg SLN dispersion, SLN incorporated hydrogel and plain Poloxamer 407 solutions were put into aluminum sample pans. An empty pan was used as a reference. Thermograms were recorded at a rate of 10 °C/min over the temperature range of 5-100 °C. Measurements were performed after preparation of SLNs and after incorporation of SLNs into copolymer solution. All samples were kept at room temperature before analysis to lose their water content.

*Determination of sol-gel transition temperature*

Sol-gel transition temperature was obtained by stirring magnet bar method (30) performed over temperature range of 5 to 50 °C. Briefly, 5 mL Poloxamer solution (with or without SLN) was placed in a 20 mL glass vial (diameter: 28 and height: 57 mm) into thermostated hot/cool water bath (Dorsa, Iran) and temperature was increased slowly, while stirring at 200 rpm using a 2 mm magnetic bar. The temperature, at which the magnet bar stopped motion, was considered as the gelation temperature.

*Measurement of gel erosion rate*

Poloxamer 407 gels undergo a process of dissolution (erosion) when come in contact (at their application site) with aqueous physiological fluids (*e.g*., tears, vaginal fluids, and extracellular fluids). This erosion rate is very important in the total release rate of the drug from the formulation. The erosion rate of prepared gels (with or without SLN) were measured here by a gravimetric method (31). In this method, the amount of dried eroded (dissolved) gel is determined gravimetrically after sampling. The slope of cumulative amount of dissolved polymer against time represents the rate of gel erosion. 

*Rheological behavior of SLN sol-gel systems*

The rheological properties of the in-situ forming systems were measured with Anton Paar Physica MCR51 rheometer (Anton Paar Company, Austria) after and before SLN incorporation in Poloxamer solution over a temperature range of 10 to 50 °C. Temperature was controlled by a circulating water bath (ViscothermVT2). Assessment of viscosity at different temperatures was done by steady shear softening/curing/gelling/hardening/temperature test. In oscillation mode, mean oscillatory parameters (storage modulus G′ and loss modulus G″ and lag phase) were calculated and evaluated as a function of the temperature over temperature range of 10-45 °C (to cover cool temperature, ambient condition and body temperature) at a heating rate of 0.2 °C min^−1^. Oscillation measurements were conducted at a reference angular frequency of 1Hz in nondestructive linear viscoelastic range and at strain amplitude of 60 N/m^2 ^using a 40 mm concentric cylinder with 2° cone angel rotational apparatus (32). Calculations were performed using Rheopuls/32V, 2.81 software.

*Statistics*

Data are reported as mean ± SD values of three different experiments. Statistical comparisons were made by unpaired Student t-test using Microsoft Excel 2010 and IBM SPSS statistics 20. P-values of < 0.05 were considered statistically significant.

## Results and Discussion

*Size, zeta potential and lipid recovery*

**Figure 1:**
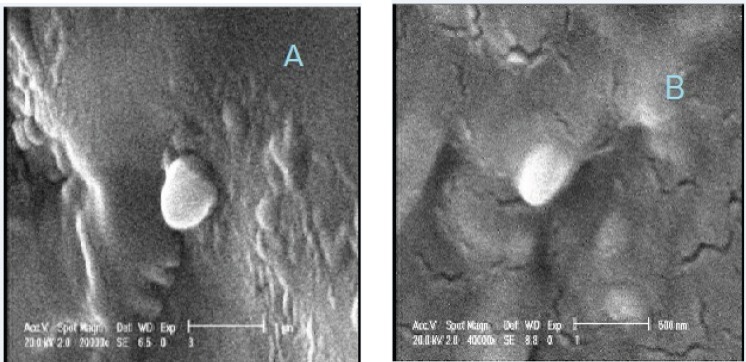
Scanning electron micrograph of freshly prepared plain solid lipid nanoparticle (A) and nanoparticles incorporated into hydrogel (B).

SLN particles showed a size of 130 ± 1.39 nm (mean ± SD, n=3) and their surfaces carried a negative charges with a zeta potential of – 44 ± 2.1 mV (mean ± SD, n=3). Under SEM, a dense solid sphere was observed for freshly prepared SLNs (Figure 1). Size increase in SLNs was observed in SEM investigation due to partial melting of some nanoparticles and aggregation of them.

Phospholipid content of SLN was calculated based on calibration curve to be 0.045 ± 0.6 mg per one mL of SLN dispersion, indicating a recovery around 91%. Our results also showed that phospholipid content does not change (P ˃ 0.05) after purification by centrifugal filtration by Vivaspin 2, MWCO: 5000.

*Gel capacity and SLN integrity in Poloxamer gel*

One of the most important factors in ability of Poloxamer 407 to form a gel at physiological temperature is its concentration. Based on previous studies (-), it is essential to choose a minimum concentration of polymer at which it forms a gel (at body temperature) to avoid side effects. Poloxamer concentration of 20 % (w/v) was selected for plain (without SLN) system here, based on the manufacturer suggestion (33). Maximum capacity of gel for incorporation of SLNs was evaluated through gel consistency and gel clarity studies on SLN-containing systems at 37 °C, as described above. Results (Table 1 ) showed that up to 50% of water can be replaced with SLN dispersion with minimum changes in clarity or consistency of the obtained gel at 20 % Poloxamer.

**Table 1:** Gel forming ability of Poloxamer 407 at 37 °C after partial replacement of pure water with SLN dispersion. Final Poloxamer concentration is 20% (w/v) in all systems

SLN dispersion (mL)	Cold water (mL)	Gel clarity*	Gel consistency*
**0**	80	+	+
**20**	60	+	+
**30**	50	+	+
**40**	40	+	+
**50**	30	+	+/-
**60**	20	_	_
**80**	0	_	_

* : +: Acceptable; +/-: Moderate;-: Not acceptable

The integrity of SLNs incorporated into sol-gel system was evaluated by scanning electron microscopy. Results (Figure 1) showed that particles keep their spherical morphology in hydrogel system. Moreover, particle size of SLN-containing gels after gel erosion at different time intervals (Table 2) increased slightly from 130 ± 1.39 in freshly prepared free SLN dispersion to 138 ± 1.82 (after 4 h) and 142 ± 2.1 (after 8 h) (P˂0.05). There were no differences between sizes of SLNs at 4 h and 8 h after gel erosion. Changes in particle size might be related to changes in hydration shell due to polymer accumulation on the surface of particles. Plus, obtained results indicated that SLN particles stay intact in the gel and are released from the Poloxamer hydrogel as particles.

**Table 2:** Particle size (nm) of plain SLNs and SLN particle released from gel over different erosion times. Data are Mean ± SD, n=3.

**Plain SLN**	Erosion time (h)	
	4 h	8 h
**130 ± 1.39**	138±1.82	142 ± 2.1

Different mechanisms have been suggested for gelation of Poloxamer 407 such as ordering of micelles to a cubic structure, dehydration of poly (propylene oxide) (PO) core, and micelles entanglements (37, 38). It is generally accepted that spherical micelles in Poloxamer 407 solution consist of a PO core with an poly (ethylene oxide) (EO) water swollen shell based on their lipophilicity (35) and proportion of EO which is approximately 70 % by weight (39). As a result, SLN would be loaded in PO core to thermodynamically stabilize the combined system.

*Differential scanning calorimetry (DSC)*

**Figure 2:**
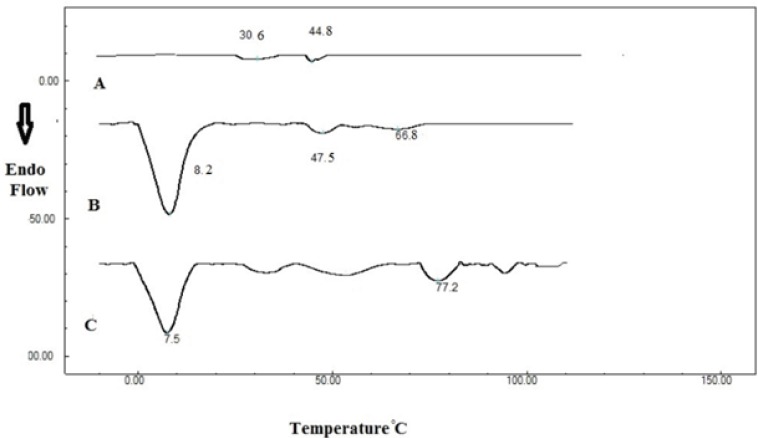
Differential scanning calorimetry thermograms of A: Poloxamer 407 (20% w/v), B: Poloxamer 407 (20% w/v) containing 20 mL SLN dispersion and C: SLN dispersion

Figure 2  shows DSC thermograms of dried 20 % Poloxamer 407 solution, indicating a transition at 30.6 °C. Addition of SLNs changed this transition temperature from 30.6 to 28.5 °C. This peak has a low enthalpy and can be detected at low scanning rates such as 0.5 °C/min (40). Enthalpy of thermogelation is reported to be very low (41). Therefore the sol-gel transition of Poloxamer 407 was determined here by the stirring method and rheological study, as described later. 

Second transition temperature in Poloxamer 407 thermogram was measured to be 44.8 and 47.5 °C before and after SLN incorporation respectively. These transitions might be related to Poloxamer melting point, which is stated to be around 50 °C (39). Another transition temperature (66.8 °C) was also observed in SLN-containing system which was attributed to stearic acid melting which is in the range of 65 to 70 °C (42).

A high-enthalpy transition was also observed in SLN system around 8 °C that was not affected by Poloxamer 407. 

*Sol-gel transition temperature*

Stirring magnet bar studies showed that Poloxamer 20% solution (sol) has a sol-gel transition temperature of 24 ± 1.2 °C. After SLN incorporation, gelling temperature increased to 27 ± 0.9 °C, which is closer to body temperature. Higher gelling temperature results in lower amount of polymer required for gel formation *in-vivo *(43).

*Erosion test*

**Figure 3:**
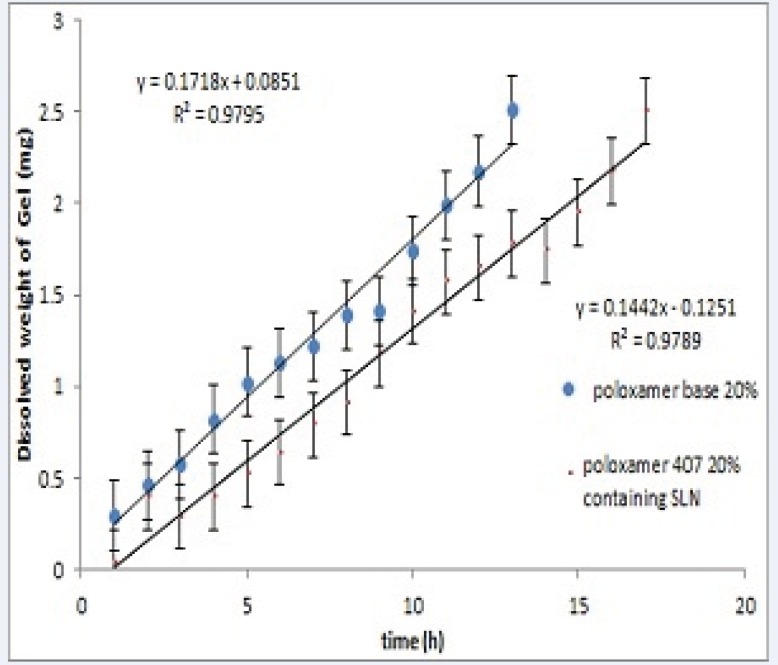
*In-vitro* erosion profile of in-situ forming systems

Figure 3 shows the erosion (dissolution) of Poloxamer gel for plain and SLN-containing systems over 20 hours. Both systems showed a linear profile of amount dissolved versus time (R^2^> 0.98). Results also show that incorporation of SLNs into Poloxamer solution decreases polymer erosion rate from 0.17 to 0.14 mg/h (P<0.05).

*Rheological properties of sol-gel systems*

**Figure 4:**
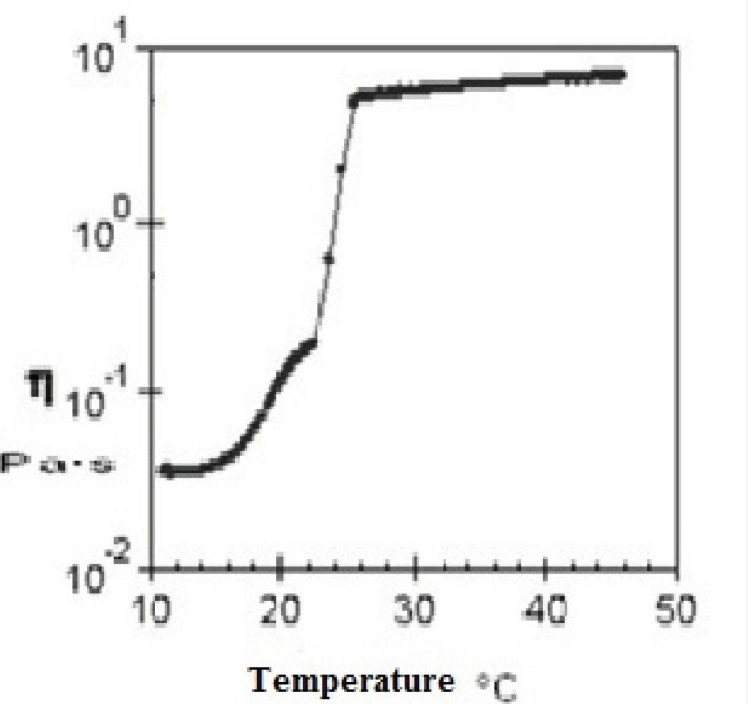
Relationship between the viscosity of a 20% (w/v) solution of Poloxamer 407 and temperature

**Figure 5:**
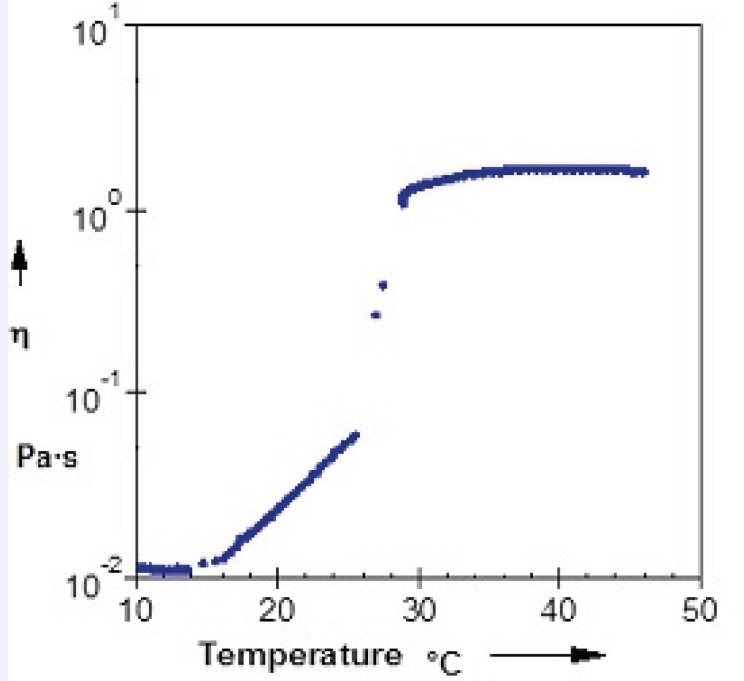
Relationship between the viscosity and temperature for 20% (w/v) solution of Poloxamer 407 containing 20 mL SLN dispersion

Evaluation of rheological properties is very useful in physicochemical characterization of gel formulations and help understanding their process of thermal gelling in-situ. Figure 4 and Figure 5 show viscosity of Poloxamer 407 solutions as a function of temperatures for plain and SLN-containing systems respectively. Both systems showed a constant viscosity upto about 15 °C, but their viscosities increased as the temperature elevated. After gel formation (sol-gel transition) viscosity became constant again. Based on these results, the sol-gel transition temperature of plain and SLN-containing system, which is equal to maximum viscosity of the system, was measured to be 26 °C and 29 °C respectively which shows good agreement with stirring test results.

The viscosity of Poloxamer 407 aqueous solutions (15–30%) against temperatures (15-35 °C) has been investigated and it has been found that an exponential relationship exists between viscosity and temperature, with the slopes depending on Poloxamer concentration (44). It has also been shown that while a sol solution of Poloxamer 407 shows a Newtonian behavior (linear relationship between shear rate and shear stress), the system changes to a gel with non-Newtonian behavior when concentration or temperature is increased (45).

Poloxamer 407 gels can show viscoelastic properties. They have an elastic or storage modulus, G', which represents the amount of energy stored and recovered per cycle of deformation of solid-like component of material. The viscous or loss modulus, G", is characteristic of liquid part (46). The most common way to determine viscoelastic properties of dispersion is oscillatory rheological study as a function of temperature within the linear viscoelastic region under shear stresses which are tolerable for gel structure.

**Figure 6:**
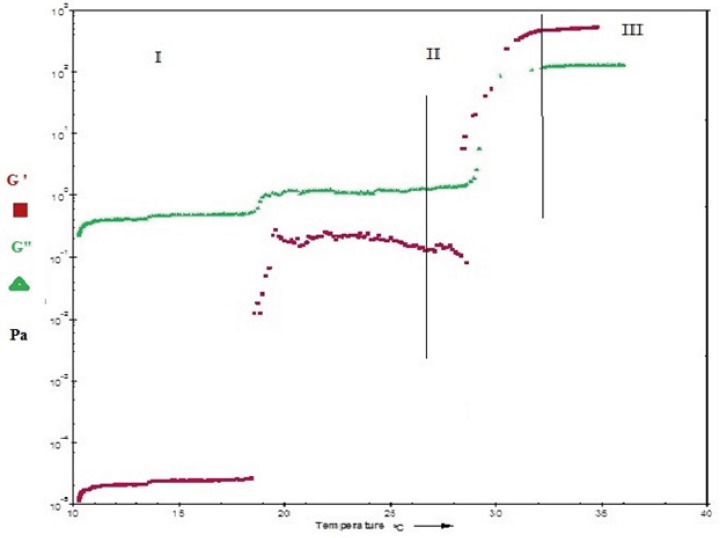
Temperature dependency of the dynamic moduli, G" and G' of a freshly prepared 20% (w/v) Poloxamer 407 solution.

**Figure 7:**
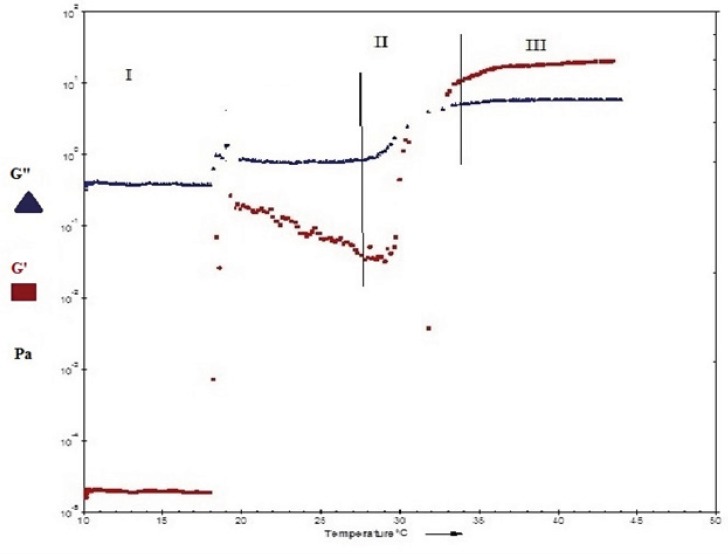
Temperature dependency of the dynamic moduli, G" and G' of 20% (w/v) Poloxamer 407 containing 20 ml SLN dispersion.

Figure 6 and Figure 7show the profile of oscillatory parameters versus temperature for plain Poloxamer and SLN-included Poloxamer systems respectively. This profile shows three distinct phases of sol, gel, gel stabilization; designated as phases I, II and III here. In phase I, that is prior to the gelling point, the elastic modulus, G', shows low values and the samples are characterized as a viscous sol system with higher G". In the second phase and at gelling temperature (phase II), drastic increase in elastic modulus, G', was observed indicating formation of gels with elastic behavior. Figures 6 and 7 show that while G' is lower than G" in phases I and II, it becomes higher than G" in later stages of phase II and phase III. G'>G" is said to be indicator of a well-built structure of a soft gel, possibly due to physical entanglements of polymeric chains (47). Final phase (phase 3) states the stabilization of the elastic modulus, G', above the transition temperature.

Our results also reveal that both plain and SLN-containing systems show similar behavior in terms of changes in G' and G" against temperature, indicating that SLN incorporation at low concentrations does not change the gel structure.

The gelation temperature (sol-gel transition) can be calculated from G′ and G" changes versus temperature and it is considered to be the temperature at which storage (G′) and loss modulus 

(G″) become equal, *i.e*. the crossover point of G′ and G″, that is usually halfway in phase II (21,48, 49). By evaluating crossing point of G′ and G″ in Figure 6 and Figure 7, the gelation temperatures were calculated to be approximately 26 ± 0.8 °C for plain Poloxamer and 29 ± 0.7 °C for SLN- containing system. These results are in agreement with stirring magnetic bar results. In this direction, it has been shown that inclusion of cyclodextrin in Poloxamer 407 gel increases sol gel transition temperature, possibly by disturbing the micellar packing and entanglements of Poloxamer 407 (30, 43).

The gelation temperature has been considered to be suitable in the range of 25-37 °C for in-situ forming gels. Lower gelling point causes gelling at normal room or laboratory conditions and causes difficulties in manufacturing, handling and administrating (50) especially in injectable formulations (51).

Another considerable parameter in injectable in-situ forming systems is gelation time or kinetic of gelation. This is the time required for an injectable in-situ forming system to turn from liquid state to gel (52) and it is equal to the time that take for the storage modulus (G′) to became higher than the loss modulus (G") in oscillatory parameters versus temperature diagrams (21, 53). In this study, as is seen in Figure 6 and Figure 7, gelling time is short (approximately one minute) in Poloxamer 407 solution and does not change after incorporation of SLN into this system. Such a fast gelation time reduces the risk of burst release due to lower possibility of dilution and drainage at the site of application (53, 54). 

**Figure 8:**
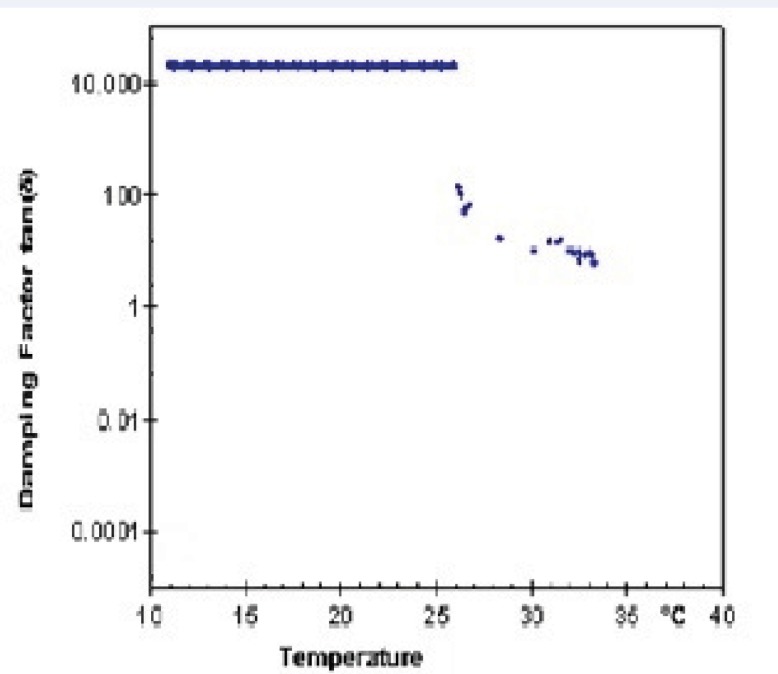
Temperature dependency of damping factor (G"/G') of 20% (w/v) freshly prepared Poloxamer 407 solution.

**Figure 9:**
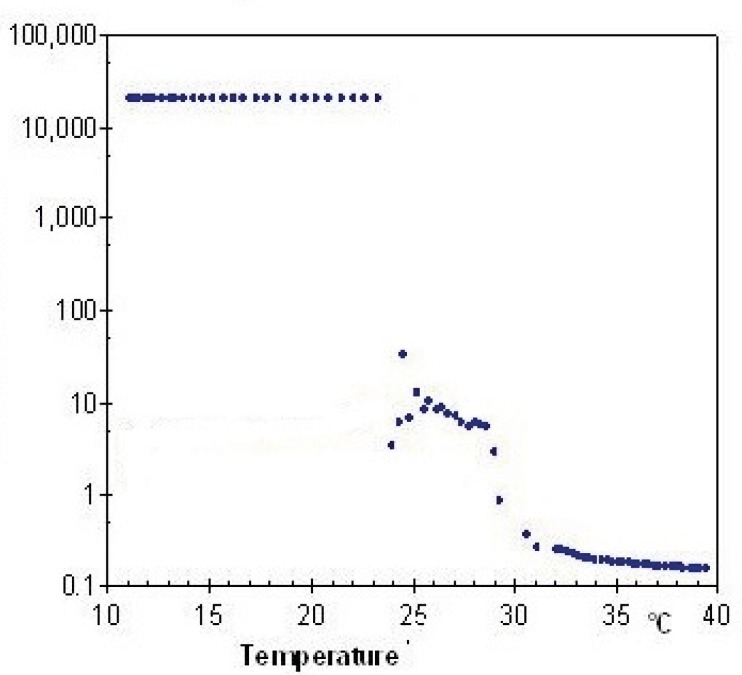
Temperature dependency of damping factor (G"/G') of 20% (w/v) Poloxamer 407 system congaing 20 mL SLN dispersion.

Lag phase or damping factor is obtained by dividing G" to G' and is an indicator of stability of polymeric structure based on their interactions. Figure 8 and Figure 9 show damping factor changes as a function of temperature in Poloxamer and combined system with nanoparticles. At low temperatures, the measured damping factor of all samples was higher than one which represents smaller interaction of internal structure in sol state. By elevation of temperature, the damping factors decreased to values less than one which shows stronger chain interaction in gel and confirm elastic behavior of gel formation. SLN incorporation decreased the damping factor (Figures 8 and 9), indicating a more stable gel.

## Conclusion

The present investigation tried to prepare a SLN-containing sol-gel system for controlled nanoparticle delivery using Poloxamer 407 system. The properties of the resultant system demonstrated that incorporation of SLN increases sol-gel transition temperature of the product. This is considered a favorable change as results in decreased polymer concentration. SLN moves the transition temperature closer to body temperature as well. This provides a wider temperature range for system handling before injection. In addition, SLN seems to decrease gel erosion rate, which is again a favorable change in terms of duration of action. Finally, gelling kinetic did not change significantly after addition of SLN and damping factor < 1 indicated stability of the SLN-containing system.

The present data show that SLNs can be incorporated into Poloxamer 407 sol-gel systems to deliver their payload in a controlled release manner. Further studies are in progress in our laboratories in order to evaluate the SLN-containing sol-gel system *in-vivo*.
